# Shikonin Binds and Represses PPARγ Activity by Releasing Coactivators and Modulating Histone Methylation Codes

**DOI:** 10.3390/nu15071797

**Published:** 2023-04-06

**Authors:** Ui-Hyun Park, HyeSook Youn, Eun-Joo Kim, Soo-Jong Um

**Affiliations:** 1 Department of Integrative Bioscience and Biotechnology, Sejong University, 209 Neungdong-ro, Gwangjin-gu, Seoul 05006, Republic of Korea; 2 Department of Molecular Biology, Dankook University, Cheonan-si 31116, Republic of Korea

**Keywords:** shikonin, adipogenesis, antagonist, epigenetic regulation, histone methylation

## Abstract

Shikonin, a natural ingredient produced by *Lithospermum erythrorhizon*, has anti-inflammatory, anti-cancer, and anti-obesity effects. It also inhibits adipocyte differentiation; however, the underlying molecular and epigenetic mechanisms remain unclear. We performed RNA-sequencing of shikonin-treated 3T3-L1 cells. Gene ontology and gene set enrichment analysis showed that shikonin is significantly associated with genes related to adipogenesis, histone modification, and PPARγ. Shikonin treatment downregulated the mRNA expression of PPARγ-responsive genes and rosiglitazone-induced transcriptional activity of PPARγ. Microscale thermophoresis assays showed a K_D_ value 1.4 ± 0.13 μM for binding between shikonin and PPARγ. Glutathione S-transferase pull-down assays exhibited that shikonin blocked the rosiglitazone-dependent association of PPARγ with its coactivator CBP. In addition, shikonin decreased the enrichment of the active histone code H3K4me3 and increased the repressive code H3K27me3 of PPARγ target promoters. Shikonin is a PPARγ antagonist that suppresses adipogenesis by regulating the enrichment of histone codes during adipogenesis. Therefore, it may be used to treat obesity-related disorders via epigenetic changes.

## 1. Introduction

The prevalence of obesity is increasing worldwide, and more than 1 billion people were considered obese in 2022. Obesity affects multiple metabolic systems and is associated with several chronic diseases, such as diabetes, hypertension, stroke, and cardiovascular disease [1]. The pathogenesis of obesity involves energy redundancy and consequent excessive white adipose tissue deposition. Adipocyte hypertrophy and hyperplasia increases white adipose tissue mass, leading to obesity [2]. Therefore, obesity may be treated by regulating adipogenesis.

Recent research has indicated that epigenetic alterations in offspring are closely linked to parental obesity and metabolic dysfunction [3,4]. Furthermore, research conducted on adipocytes, animals, and genome-wide genomics has demonstrated that epigenetic modifications can regulate adipogenesis [5,6,7]. Epigenetic alterations involve the regulation of gene activity without changes in the DNA sequence. Major epigenetic alterations include chromatin remodeling, DNA methylation, microRNA changes, and histone modifications, which dynamically control gene expression by altering target gene promoters [3,8]. Histone modifications are one of the key epigenetic alterations that govern the expression of target genes, as demonstrated in various studies. Enrichment of active histone codes on target promoters, such as acetylation at lysine 9 in histone H3 (H3K9Ac), tri-methylation at lysine 4 in histone H3 (H3K4me3), and H3K36me3, upregulates the expression of target genes. Conversely, deposition of repressive histone codes on target promoters, such as H3K9me3 and H3K27me3, downregulates the expression of target genes. These modifications are regulated by the recruitment of histone lysine methyltransferases or histone lysine demethylases on target promoters [8,9].

Adipogenesis maintains energy balances in mammals and is mainly controlled by adipogenic transcription factors, such as the sterol regulatory element binding protein family, CCAAT-enhancer binding protein family, and peroxisome proliferator-activated receptor-γ (PPARγ) [10]. Despite extensive study on the functions of these transcription factors in adipogenesis, the precise effects of epigenetic regulation on this process remain poorly understood. However, other studies have documented that certain dietary components have the ability to induce epigenetic modifications that reduce adipogenesis [6,7].

Shikonin is a purple derivative of the naphthoquinone pigment found in the root of *Lithospermum erythrorhizon*, a traditional herbal medicine in China and Korea [11,12]. Shikonin has beneficial effects on inflammation [11], breast cancer [13], hepatic steatosis [14], and obesity [15]; however, the effects of shikonin on epigenetic alterations in adipogenesis have not been investigated.

We explored the epigenetic alterations caused by shikonin during adipogenesis. Genome-wide analysis of RNA-sequencing demonstrated that shikonin reduced the expression of adipogenic genes and PPARγ target genes. In addition, we determined that shikonin directly binds PPARγ and dissociates the coactivator CBP, leading to the suppression of the transcriptional activity of PPARγ. Furthermore, a chromatin immunoprecipitation (ChIP) assay exhibited that shikonin oppositely regulates the enrichment levels of H3K4me3 and H3K27me3 on promoters of PPARγ target genes. Collectively, our findings suggest that shikonin is a PPARγ antagonist and has differential enrichment effects on H3K4me3 and H3K27me3. Therefore, the epigenetic effects of shikonin may be used to treat obesity.

## 2. Materials and Methods

### 2.1. Chemicals and Reagents

We purchased 3-(4,5-dimethylthiazol-2-yl)-2,5-diphenyl tetrazolium bromide, Oil Red O (ORO), dexamethasone, 3-isobutyl-1-methylxanthine (IBMX), insulin, dimethysulfoxide (DMSO), and shikonin from Sigma-Aldrich (St. Louis, MO, USA). Bovine serum (BS) and fetal BS (FBS) were obtained from Gibco (Grand Island, NY, USA). Antibodies against trimethylated H3K4 (H3K4me3; Millipore; 04-745) and trimethylated H3K27 (H3K27me3; Millipore, 07-449) were used for experiments.

### 2.2. Adipocyte Differentiation

Briefly, 3T3-L1 preadipocyte cells were grown in DMEM supplemented with 10% (*v*/*v*) BS and 1% antibiotics/antimyotics (Invitrogen, Carlsbad, CA, USA) at 37 °C and 5% CO_2_ to achieve confluence. After 2 days, adipocyte differentiation (day 0) was induced with 0.5 μM dexamethasone, 100 μM IBMX, and 1 μg/mL insulin with or without shikonin. During the 8 days of adipogenesis and 2 days thereafter, cells were cultured in fresh DMEM containing 10% FBS, 1 μg/mL insulin, and shikonin.

### 2.3. Oil Red O (ORO) Staining

ORO staining was performed as follows. The fully differentiated 3T3-L1 cells were washed with phosphate-buffered saline (PBS; pH 7.4) and fixed with 2 mL of 10% formalin in PBS for 10 min. The cells were washed twice with distilled water and stained with 1 mL of 0.5% ORO (Sigma-Aldrich) for 10 min with gentle agitation. After removing the excess stain using 60% isopropanol, cells were rinsed with PBS and photographed. The deposited lipids were extracted with 100% isopropanol and the absorbance was measured at an optical density of 500 nm.

### 2.4. Real-Time Quantitative RT-PCR (RT-qPCR)

RT-qPCR was performed as follows. The total RNA was extracted from cells using a TRIzol reagent (Invitrogen, Carlsbad, CA, USA) according to the manufacturer’s instructions. The cDNA was synthesized with 1 μg of the total RNA using MMLV reverse transcriptase and random primers (Invitrogen). The qPCR was performed using the Icycler CFX96 real-time PCR detection system (Bio-Rad, Hercules, CA, USA) and SYBR Green PCR mixture (Toyobo Co., Ltd., Osaka, Japan). The primers used for qPCR are shown in Supplementary Table S1. The expression levels were normalized to those of the internal standard GAPDH. The expression level was expressed as the relative change to controls.

### 2.5. RNA-Sequencing and Gene Ontology (GO) Analysis

Briefly, total RNA was extracted from DMSO- and shikonin-treated 3T3-L1 cells after differentiation and transferred to e-Biogen (Seoul, Republic of Korea) to check RNA quantity and quality, construct an mRNA-seq library, and detect differentially expressed genes. Generated Bedgraph files were uploaded and presented in the University of California Santa Cruz Genome Browser (http://genome.ucsc.edugsea, accessed on 27 June 2019), as described previously [6,7]. The results were filtered based on a cut-off of a 1.5-fold difference. Clustering analysis was performed, and a heat map was generated using Multiple Experiment Viewer software (MeV 4.9.0). Differentially expressed genes were analyzed using GO (http://www.geneontology.orggsea, accessed on 27 June 2019) and MSigDB software (version 5.2; http://software.broadinstitute.org/gsea/msigdbgsea, accessed on 27 June 2019).

### 2.6. Gene Set Enrichment Analysis (GSEA)

GSEA was performed as described previously (http://www.broadinstitute.org/gsea, accessed on 27 June 2019) [7]. To analyze the RNA-seq data, we normalized the expression levels and ranked them using a signal-to-noise metric. We measured enrichment scores (ESs) by performing 1000 permutations of random gene sets. The normalized enrichment score (NES) was then calculated by dividing the ES by the mean permutation of ES. Gene sets were added to the gene set file msigdb.v5.1.symbols.gmt for GSEA. A nominal *p*-value < 0.05 and a false discovery rate (FDR) of 0.25 were considered statistically significant.

### 2.7. Luciferase Reporter Gene Assays

Reporter gene assays were performed as described previously [16]. HEK293 cells were seeded in 12-well plates and co-transfected with PPARγ, a PPRE-tk-luciferase reporter gene, and SV-40-driven β-galactosidase expression vectors using LipofectAMINE (Invitrogen). After transfection overnight, cells were treated with DMEM containing 5% FBS stripped with charcoal and incubated overnight in the presence of rosiglitazone (0.5 μM) and increasing amounts of shikonin. Luciferase activity was measured and displayed as a fold-change relative to control.

### 2.8. Microscale Thermophoresis (MST) Analysis

MST analysis was performed as follows. A His-tag labeling kit, Red-Tris NTA (NanoTemper Technologies, Munich, Germany) was used to label the recombinant His-fusion PPARγ-ligand-binding domain (LBD) at cysteine according to the manufacturer’s protocols. Briefly, we incubated 800 nM of the protein with 0.9 equivalents of dye in PBS-T buffer (pH 7.5, 0.1% Triton X-100 and 0.5% Tween 20) for 1 h at room temperature in the absence of light. To determine the K_D_ of His-PPARγ to shikonin, 40 nM of labeled His-PPARγ was incubated with increasing concentrations of shikonin in PBS-T buffer (pH 7.5, 0.2% Triton X-100 and 0.2% Tween 20). Samples were loaded on standard glass capillaries (Monolith NT.155 Capillaries) and subjected to MST analysis using a Monolith NT.115 Pico and IR laser power of 20%. K_D_ values were calculated using NanoTemper software (MO.Affinity Analysis; version 2.2.7).

### 2.9. Glutathione-S-Transferase (GST) Pull-Down

GST pull-down assays were performed using purified proteins (GST, GST-CBP, and His-PPARγ LBD). These proteins were mixed with 50 μL of binding buffer (50 mM Tris-Cl, pH 7.5, 200 mM NaCl, 1 mM EDTA, 0.5% Triton X-100, 1 mM PMSF, 0.1% Nonidet P-40) in the presence or absence of 1 μM rosiglitazone and increasing concentrations of shikonin. After 30 min of incubation at 30 °C, 100 μL of a 50% slurry of GST beads equilibrated with binding buffer was added and incubated for 1 h at room temperature. The beads were washed three times with the binding buffer, bound proteins were eluted with SDS sample buffer by boiling for 10 min, and proteins were visualized by western blotting using an anti-His antibody.

### 2.10. ChIP Assays

ChIP assays were performed as described previously [17]. DNA pellets were subjected to qPCR analysis using primer pairs targeting specific promoters (Supplementary Table S1). Rabbit immunoglobulin G (IgG) was used as the negative control. The ratios of fold-enrichment from each antibody were calculated based on the Ct values normalized to that of IgG. The results were represented as the percentage of input.

### 2.11. Statistical Analysis

Values represent the means ± standard deviations (SDs) from at least three independent experiments. Groups were compared using paired *t*-tests. If the *p*-value was lower than 0.05 (*) or 0.01 (**), the result was considered statistically significant.

## 3. Results

### 3.1. Shikonin Inhibits Adipogenesis and Downregulates Adipogenic Genes

**Figure 1:**
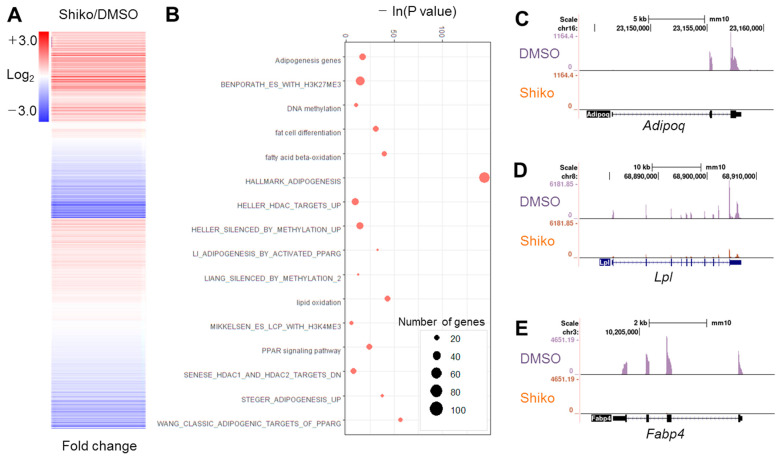
Genome-wide RNA-seq analysis. (**A**) Clustering analysis of 2406 genes with 1.5-fold changes following shikonin treatment. (**B**) Gene Ontology (GO) analysis of genes with 1.5-fold changes. (**C**–**E**) Bedgraph analysis of three adipogenic genes downregulated by shikonin treatment. Enrichment of tag fragments from RNA-seq results are shown in the gene bodies of *Fabp4* (**C**), *Adipoq* (**D**), and *Lpl* (**E**).

Shikonin inhibits fat cell differentiation [12]; however, the underlying molecular and epigenetic mechanisms remain unclear. We measured cytotoxicity of shikonin in 3T3-L1 cells. Shikonin did not show significant toxicity up to the concentration of 2 μM (Supplementary Figure S1A,B). We confirmed the antagonistic effect of shikonin on the differentiation of 3T3-L1 preadipocytes by ORO staining (Supplementary Figure S1C). Shikonin treatment reduced lipid accumulation (Supplementary Figure S1D). The mRNA expression of adipogenic genes, such as *Fabp4*, *Adipoq*, and *Lpl*, was suppressed by shikonin treatment (Supplementary Figure S1E). To elucidate the genome-wide function of shikonin, we performed RNA-seq using shikonin-treated preadipocytes. Clustering analysis exhibited that 2406 genes exhibited a change > 1.5-fold in shikonin-treated 3T3-L1 cells (Figure 1A). Of these, 933 genes were upregulated, and 1473 genes were downregulated (Supplementary Table S3). GO analysis exhibited that shikonin-regulated genes were closely related to adipogenesis, DNA methylation, lipid oxidation, and histone methylation (Figure 1B). RNA-seq data, analyzed by the Genome Browser, indicated that shikonin decreases the accumulation of tags in the exon of three adipogenic genes, *Fabp4*, *Adipoq*, and *Lpl*, in 3T3-L1 cells (Figure 1C). GSEA also showed that shikonin-regulated genes were strongly associated with adipogenesis, lipid metabolism, histone deacetylases, bivalent histone codes, and PPARγ targets (Supplementary Figure S1F). We focused on PPARγ-related genes due to the large number of significant GO terms associated with PPARγ based on an analysis of 1473 downregulated genes (Supplementary Table S2). These results suggest that shikonin represses the mRNA expression of the PPARγ response genes required for fat deposition during adipogenesis of 3T3-L1 cells.

### 3.2. Shikonin Reduces the Expression of PPARγ Target Genes

**Figure 2:**
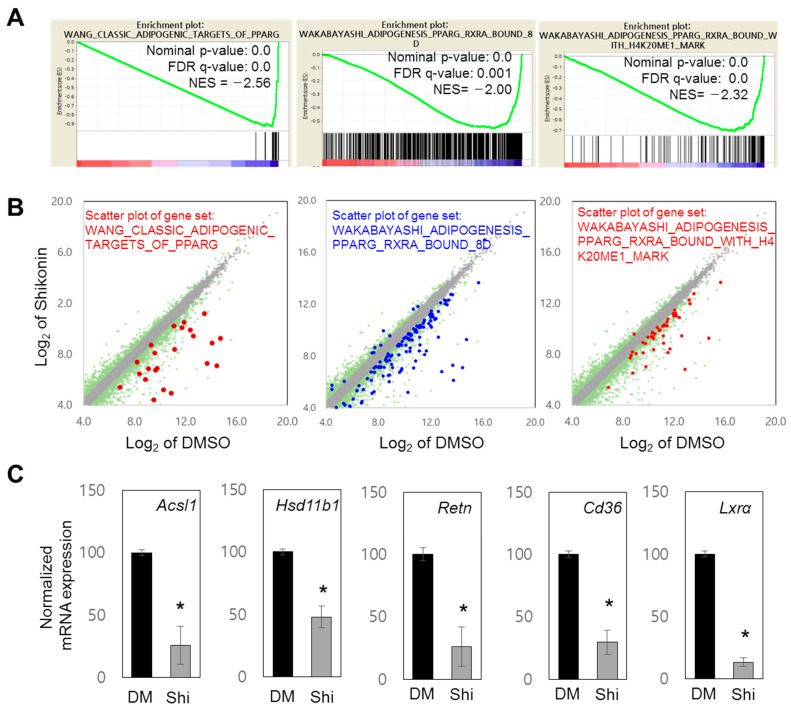
Genome-wide RNA-seq analysis of shikonin-regulated genes. (**A**) Gene sets significantly associated with PPARγ were targeted through GSEA, and the normalized enrichment score (NES) was measured. Gene sets with a nominal *p*-value of <0.05 and false discovery rate (FDR) *q*-value of <0.25 were considered significantly enriched. (**B**) Scatter plot analysis of selected gene sets. In total, 13,965 significant genes (log2 value of mRNA expression > 4.0 in RNA-seq of DMSO or shikonin) are displayed in gray. Among them, 2406 genes with a 1.5-fold change in expression level are shown in green. The distribution of genes associated with the indicated GO terms are displayed by brown, blue, and red spots. (**C**) Effects of shikonin on the mRNA expression of five PPARγ response genes. 3T3-L1 cells were differentiated and treated with DMSO or shikonin, then their transcript expressions were measured by RT-qPCR and normalized to GAPDH. Results are presented as the relative expression compared to DMSO controls. Values are represented as the means ± SDs from three independent experiments (* *p* < 0.05).

In addition to GO analysis, GSEA using RNA-seq data supported the role of shikonin in the regulation of PPARγ-bound genes: WANG_CLASSIC_ADIPOGENIC_TARGETS_OF_PPARG, WAKABAYASHI_ADIPOGENESIS_PPARG_RXRA_BOUND_8D, and WAKABAYASHI_ADIPOGENESIS_PPARG_RXRA_BOUND_WITH_H4K20ME1_MARK (Figure 2A, Supplementary Figure S2A). The PPARγ-related gene sets showed decreased expression (Figure 2A). Scatter plot analysis also showed reduced expression of genes in each gene set (Figure 2B, Supplementary Tables S4–S6). Using previously identified PPARγ target genes, including *Acsl1*, *Hsb11b1*, and *Retn* [7], we demonstrated that these genes are repressed by shikonin (Figure 2C). The mRNA expression levels of other known target genes of PPARγ (*Glut4*, *Leptin*, *lxrb,* and *Pparg*) were significantly attenuated by shikonin (Supplementary Figure S2B). These data suggest that shikonin may inhibit the transcriptional activity of PPARγ through direct binding.

### 3.3. Shikonin Acts as an Antagonist by Directly Binding PPARγ

**Figure 3:**
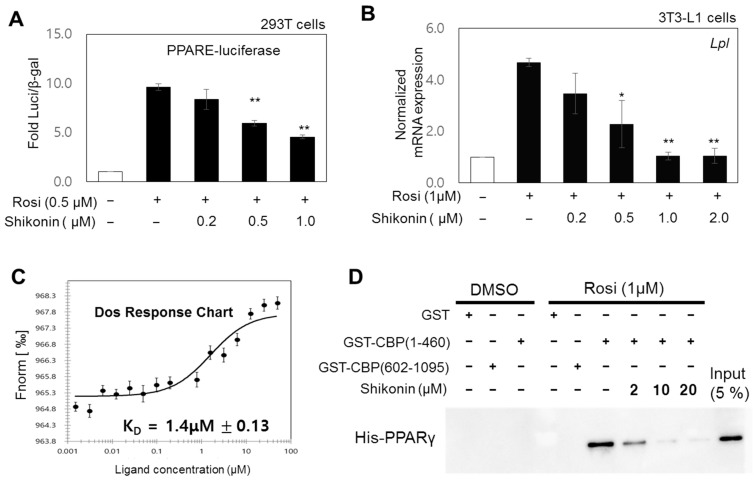
Antagonistic effects of shikonin on PPARγ regulation. (**A**) Results of the luciferase reporter gene assay. HEK293 cells were cotransfected as described in the Materials and Method section and treated with rosiglitazone (Rosi, 0.5 μM) and the indicated concentrations of shikonin. Luciferase values were normalized to the β-galactosidase activity. The error bars represent means ± SDs of three independent experiments (** *p* < 0.01). (**B**) Effects of shikonin on Rosi-induced expression of the *Lpl* gene. 3T3-L1 cells were differentiated and treated with 1 μM of Rosi and the indicated concentrations of shikonin for 6 days. Expression of mRNA, measured by RT-qPCR, was normalized to the GAPDH expression level and indicated as fold change relative to that of the DMSO control. Bars represent means ± SDs of three independent experiments (* *p* < 0.05 and ** *p* < 0.01). (**C**) Microscale thermophoresis (MST) assays were performed as described in the Materials and Methods section. (**D**) Effects of shikonin on the rosiglitazone-induced interaction of PPARγ with the coactivator CBP. GST pull-down assays were performed as described in the Materials and Methods section. The immobilized GST-CBP (amino acids 1–460 or 602–1095) fusion protein was incubated with the indicated concentration of shikonin in the presence of 1 μM of Rosi and a His-PPARγ ligand-binding domain (LBD). Bound proteins were visualized by western blotting using an anti-His antibody. The input represents 5% of His-PPARγ used for the binding assays.

Based on our results, we hypothesized that shikonin directly interacts with the ligand-binding domain of PPARγ and suppresses the transcriptional activity of PPARγ. We explored the effect of shikonin on PPARγ activity. Luciferase reporter gene assays were carried out in HEK293 cells. Cells were co-transfected with a PPARE-tk-luciferase reporter and a PPARγ-expressing plasmid in the presence of 1 μM rosiglitazone and increasing concentrations of shikonin. Shikonin inhibited the ligand-induced gene activation of PPARγ in a dose-dependent manner (Figure 3A). Next, we measured the inhibitory effects of shikonin on the ligand-induced expression of an endogenous PPARγ target gene, *Lpl* (Figure 3B). Similar results were obtained using other PPARγ target genes, including *Adipoq*, *Glut4*, and *Retn* (Supplementary Figure S3A). Protein expression of PPARγ target genes, *Lxra*, *Adipoq*, and *Pparg*, also reduced upon shikonin treatment during adipogenesis in 3T3-L1 cells (Supplementary Figure S3B). Furthermore, MST assays were used to determine whether shikonin binds to PPARγ for suppressing PPARγ activity. The binding of shikonin and PPARγ-LBD had a dissociation constant K_D_ of 1.4 ± 0.13 μM (Figure 3C), which is comparable to that for the binding of PPARγ-LBD and rosiglitazone, a synthetic PPARγ agonist (K_D_ = 0.37 ± 0.033 μM) [7]. To demonstrate the antagonistic role of shikonin, we performed GST pull-down assays using purified GST-CBP (amino acids 1–460) and His-PPARγ-LBD in the presence of rosiglitazone and increasing concentrations of shikonin. Rosiglitazone-induced binding of the coactivator CBP to PPARγ-LBD was progressively impaired with increasing shikonin concentrations (Figure 3D). These findings suggest that shikonin is an antagonist of PPARγ and competitively binds PPARγ-LBD, leading to coactivator dissociation.

### 3.4. Shikonin Inhibits PPARγ Target Gene Expression through Enrichment of Active or Repressive Histone Codes on Target Promoters

**Figure 4:**
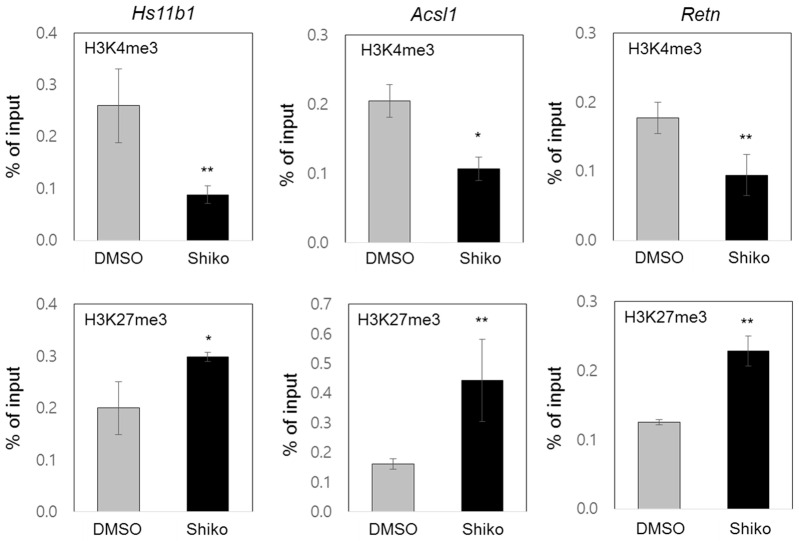
Epigenetic regulation of PPARγ target genes by shikonin. Eight days after adipogenesis, 3T3-L1 cells were fixed and harvested for ChIP assays using the indicated antibodies against H3H4me3 and H3K27me3. Promoter occupancy was determined using qPCR and primer sets on the targeted promoters of three genes (*Hs11b1*, *Acsl1*, and *Retn*). Data are represented as means ± SDs for three independent experiments (* *p* < 0.05 and ** *p* < 0.01).

GSEA and GO analyses of the genome-wide data were performed to determine the epigenetic associations of shikonin (Figure 1B and Supplementary Figure S1F). Analysis of the gene sets regulated by shikonin, including histone deacetylases, bivalent histone codes, and PPARγ targets, suggested potential changes in the histone codes of PPARγ target promoters. To investigate the differential enrichment of bivalent histone codes of PPARγ target promoters, we performed ChIP assays at the PPAREs of the PPARγ target genes *Hs11b1*, *Acsl1*, and *Retn*. Shikonin treatment decreased the accumulation of the active histone code (H3K4me3) and increased the accumulation of the repressive histone code (H3K27me3) in the upstream promoters containing PPAREs of the three genes (Figure 4). Similar results were observed for the other two PPARγ target genes, *Adipoq* and *Fabp4* (Supplementary Figure S4). Furthermore, we examined the enrichment of histone modifying enzymes such as Mll2, Ezh2, and Utx on the promoters of *Hs11b1*, *Acsl1*, and *Retn*. Enrichment of Mll2, a histone H3K4 methyltransferase, was significantly reduced on the PPARγ target promoter in shikonin-treated 3T3-L1 cells. In the presence of shikonin, the attenuated enrichments of active histone code, H3K4me3, and Mll2 were observed on the PPARγ target promoter, along with a concomitant decrease in gene expression. Upon treatment with shikonin, there was a significant increase in the recruitment of Ezh2, a histone H3K27 methyltransferase, while the recruitment of Utx, a H3K27 demethylase, was markedly decreased on the promoter of PPARγ-responsive genes. The increased recruitment of H3K27me3 and Ezh2 and the decreased recruitment of Utx on the PPARγ target promoter are consistent with conditions that repress gene expression upon shikonin treatment (Supplementary Figure S4B). Our results suggest that shikonin inhibits the expression of PPARγ response genes through the differential enrichment of bivalent histone codes and the opposite recruitment of histone modifying enzymes on target promoters, thereby inhibiting adipogenesis.

## 4. Discussion

Shikonin impairs adipocyte differentiation [12]. As a ligand-dependent transcription factor, PPARγ modulates the expression of genes associated with fat deposition during adipogenesis [10]. We investigated the pathophysiological and epigenetic mechanisms underlying shikonin-mediated inhibition of PPARγ activity. Our genome-wide RNA-seq analysis indicated that shikonin modulates the gene sets associated with adipogenesis and PPARγ target genes. We demonstrated that PPARγ target genes are downregulated by shikonin treatment during adipogenesis of 3T3-L1 cells. The reporter gene assay and RT-qPCR analysis showed that shikonin inhibits the ligand-induced PPARγ activation. Next, we evaluated whether shikonin interacts with PPARγ-LBD and antagonizes PPARγ activity. The MST analysis showed that the dissociation constant, K_D_ for the binding of shikonin to PPARγ-LBD was 1.4 μM, compared to the K_D_ of 0.37 μM for rosiglitazone, a synthetic selective PPARγ agonist. Furthermore, the competitive assays indicated that the rogilitazone-induced interaction between the coactivator CBP and PPARγ was significantly disrupted in the presence of shikonin, suggesting an antagonistic role of shikonin on PPARγ transcriptional activity. In addition, our RNA-seq analysis revealed the possible involvement of shikonin in regulating gene sets associated with epigenetics, including histone deacetylases, DNA methylation, histone methylation, and bivalent histone codes (H3K4me3 and H3K27me3). ChIP assays of PPARγ target genes showed that shikonin reduced the abundance of the active histone code (H3K4me3) and increased the abundance of the repressive histone code (H3K27me3) in the promoter regions of the PPAREs of genes, leading to reduced PPARγ transcriptional activity. These abundance of H3K4me3 or H3K27me3 were regulated by histone methyltransferase and demethylase. Through additional ChIP assays, we found that Mll2, which increases the abundance of the active histone code H3K4me3 in PPARE, are recruited to the PPARγ target promoter [6]. Shikonin was shown to reduce the recruitment of Mll2 to PPARE, resulting in a decrease in the level of H3K4me3. This indicates that PPARγ, by binding to shikonin, is unable to recruit co-activators. Also, we found that under shikonin-treated conditions, the occupancy of Ezh2 and Utx increased and decreased in the PPARE of the PPARγ target promoter, respectively. Enrichment of H3K27me3, as a repressive histone code, increases due to Ezh2-mediated methylation and decreases due to Utx-mediated demethylation. This suggests that the binding between PPARγ and shikonin induces the recruitment of Ezh2 instead of Utx, thus resulting in an increased repressive histone code and consequently inhibiting gene expression. Although other epigenetic alterations should be evaluated in future studies, our ChIP data support the epigenetic role of sikonin in suppressing PPARγ-responsive genes through differential accumulation of bivalent histone codes, which is regulated by histone modifying enzymes.

Obesity increases the risk of metabolic disorders such as hyperlipidemia, hyperglycemia, hypercholesterolemia, and diabetes. Thiazolidinediones (TZDs), such as troglitazone, pioglitazone, and rosiglitazone, are mainly used for the treatment of diabetes. They act by decreasing insulin resistance and enhancing insulin sensitivity [18]. Troglitazone was approved by the U.S. Food and Drug Administration in 1997, but was withdrawn from the market because of serious adverse effects [19]. PPARγ, which is highly expressed in adipose tissue, plays a pivotal role in adipocyte differentiation through regulation of adipogenic gene expression and control of glucose homeostasis and insulin sensitivity. Because TZD binding to PPARγ-LBD increases the transcriptional activity of PPARγ, several ongoing studies are investigating therapeutic agents that target PPARγ [20]. Recent studies have shown that certain synthetic and natural substances that inhibit PPARγ are effective in treating obesity [20,21].

Shikonin is a natural component found in *Lithospermum erythrorhizon* and has been used as one of the herbal medicines in Asia [12]. We showed that shikonin acts as an antagonist of PPARγ and inhibits PPARγ transcriptional activity. The K_D_ of binding of shikonin to PPARγ-LBD was 1.4 μM, suggesting that shikonin is a strong antagonist that competes with rosiglitazone (0.37 μM for PPARγ). Importantly, shikonin is a natural substance and has been used in medicines and food. Animal experiments have demonstrated the anti-obesity effects of the natural components of dietary plants [22,23,24]. Shikonin has also been shown to effectively alleviate obesity by regulating lipid metabolism in adipose and liver tissues [25]. Additionally, shikonin derivatives can be used in the treatment of cancer, either alone or in combination with anti-cancer drugs, due to their synergistic interaction [26]. These derivatives are likely to bind to PPARγ, similar to shikonin. As a result, a variety of shikonin derivatives can be used to treat various diseases, including cancer and metabolic disfunction. Most of all, unlike TZD, which has multiple side effects, shikonin is safe as a natural component. Therefore, its derivatives may be effective against metabolic diseases such as obesity and diabetes.

## Data Availability

The data presented in this study are available on request from the corresponding author. Our RNA-seq data will be deposited in NCBI’s Gene Expression Omnibus (GEO).

## Supplementary Materials

The following supporting materials can be downloaded at: https://www.mdpi.com/article/10.3390/nu15071797/s1. Supplementary Table S1: Primer sequences used for qPCR. Supplementary Table S2: PPARγ-related terms found in GO analysis using 1473 down-regulated genes. Supplementary Table S3: Genes changed 1.5-fold in shikonin treated 3T3-L1 cells. Supplementary Table S4: Genes associated with WAKABAYASHI_ADIPOGENESIS_PPARG_RXRA_BOUND_8D. Supplementary Table S5: Genes associated with WAKABAYASHI_ADIPOGENESIS_PPARG_RXRA_BOUND_WITH_H4K20ME1_MARK. Supplementary Table S6: Genes associated with WANG_CLASSIC_ADIPOGENIC_TARGETS_OF_PPARG. Supplementary Figure S1: Anti-adipogenic effects of shikonin in 3T3-L1 cells. Supplementary Figure S2: Validation of RNA-seq data. Supplementary Figure S3: Effect of shikonin on rosiglitazone-induced activation of the PPARγ target genes. Supplementary Figure S4: Epigenetic regulation of PPARγ target genes by shikonin.
