# Supplementary material for: Shikonin Binds and Represses PPARγ Activity by Releasing Coactivators and Modulating Histone Methylation Codes
Source: Nutrients. 2023 Apr 6;15(7):1797. doi: 10.3390/nu15071797 (PMC10097191; doi:10.3390/nu15071797)
Supplement: Supplementary file 1 [file nutrients-15-01797-s001.zip › nutrients-2288296-supplementary.pdf]

Shikonin binds and represses PPARγ activity by releasing coactivator and modulating histone methylation codes

Ui-Hyun Park [1], Jin-Taek Hwang[2], HyeSook Youn[1], Eun-Joo Kim[3], and Soo-Jong Um[1,*]

[1] Department of Integrative Bioscience and Biotechnology, Sejong University, 209 Neungdong-ro, Gwangjin-gu, Seoul 05006, Republic of Korea

[2] Department of Molecular Biology, Dankook University, Cheonan-si 31116, Republic of Korea

*Correspondence to Soo-Jong Um:

E-mail: umsj@sejong.ac.kr; Tel.: +82-2-3408-3641; Fax: +82-2-3408-4334

Supplementary Table S1. Primer sequences used for qPCR.

Gene	Forward (5'-3')	Reverse (5'-3')
ChIP-qPCR:		
Adipoq	CCTGTTTCCAGGCTTTGGCC	GGTGCTGGGAATTGAACTCA
Fabp4	ATGCCCTGACCATGTGA	AAATTCAGAAGAAAGTAAACACAT TATT
Lpl	CCACTGCACAGCTGTTTAAGTGACTGG	CCTCCCGGTAGGCAAACTGG
Retn	GCTACAAGGCTACCCTCAGC	GGGGAGGTTTGGAGGTGATG
Hsd11b1	GGAACAATGGGCCCCAAAAC	CTGGCCTCCTTTTCTCAGCA
Acsl1	CGGTGGGGTGACTCTACTCT	TCACATGCCGAGTCCTACAC
RT-qPCR:		
Adipoq	TGACGACACCAAAAGGGCTC	CACAAGTTCCCTTGGGTGGA
Fabp4	AAAGACAGCTCCTCCTCGAAGGTT	TGACCAAATCCCCATTTACGC
Lpl	ATCCATGGATGGACGGTAACG	CTGGATCCCAATACTTCGACCA
Retn	CAGAAGGCACAGCAGTCTTG	ACGCTCACTTCCCCGACAT
Acsl1	GCCGCGACTCCTTAAATAGC	GTTCTCTATGCAGAATTCTCCT CC
Hsd11b1	CCTGCCTGGGAGGTTGTAGA	TCCCTGGAGCATTTCTGGTC
CD36	GTCCTGGCTGTGTTTGGAGG	CTTGGCTAGATAACGAACTCTGTA
Glut4	AGGGGCCTGCCCGAAAGAGT	CTGTTGGCTCAGCTGCAGCA
Leptin	CCTCACCAGCCTGCCTTCCCA	TCCAGGACGCCATCCAGGCT
Lxra	GCTGCCCAGCAACAGTGTAA	CTGCCGGGGTTGTACCTCCGT
Lxrb	GGCTTCTTCCGGCGCAGTGT	CCTTCCCCGGAGCCCTGGCT

Supplementary Table S2. PPARγ related terms found in GO analysis using 1473 down-regulated genes.

ln(P)	Term	GO Tree
-56.15	WANG_CLASSIC_ADIPOGENIC_TARGETS_OF_PPARG	MSigDB lists
-53.82	WAKABAYASHI_ADIPOGENESIS_PPARG_RXRA_BOUND_8D	MSigDB lists
-43.29	WAKABAYASHI_ADIPOGENESIS_PPARG_RXRA_BOUND_WITH_H4K20ME1_MARK	MSigDB lists
-32.26	LI_ADIPOGENESIS_BY_ACTIVATED_PPARG	MSigDB lists
-24.09	PPAR signaling pathway	KEGG pathways
-24.08	KEGG_PPAR_SIGNALING_PATHWAY	MSigDB lists
-20.1	PPAR signaling pathway	WikiPathways
-17.97	WAKABAYASHI_ADIPOGENESIS_PPARG_RXRA_BOUND_36HR	MSigDB lists
-16.09	GSE37533_PPARG1_FOXP3_VS_FOXP3_TRANSDUCED_CD4_TCELL_DN	MSigDB lists
-15.86	GSE37533_PPARG2_FOXP3_VS_FOXP3_TRANSDUCED_CD4_TCELL_DN	MSigDB lists
-13.93	WAKABAYASHI_ADIPOGENESIS_PPARG_BOUND_8D	MSigDB lists
-12.67	GSE37534_UNTREATED_VS_PIOGLITAZONE_TREATED_CD4_TCELL_PPARG1_AND_FOXP3_TRASDUCED_DN	MSigDB lists
-12.54	GSE37533_PPARG1_FOXP3_VS_PPARG2_FOXP3_TRANSDUCED_CD4_TCELL_PIOGLITAZONE_TREATED_DN	MSigDB lists
-8.72	V$PPAR_DR1_Q2	MSigDB lists
-8.43	GSE37533_PPARG1_FOXP3_VS_FOXP3_TRANSDUCED_CD4_TCELL_PIOGLITAZONE_TREATED_UP	MSigDB lists
-7.51	GSE5679_CTRL_VS_PPARG_LIGAND_ROSIGLITAZONE_AND_RARA_AGONIST_AM580_TREATED_DC_UP	MSigDB lists
-5.00	GSE37533_UNTREATED_VS_PIOGLIZATONE_TREATED_CD4_TCELL_PPARG1_AND_FOXP3_TRASDUCED_UP	MSigDB lists
-4.93	GSE25123_WT_VS_PPARG_KO_MACROPHAGE_IL4_AND_ROSIGLITAZONE_STIM_UP	MSigDB lists
-4.64	GSE25123_CTRL_VS_ROSIGLITAZONE_STIM_PPARG_KO_MACROPHAGE_UP	MSigDB lists
-4.63	GSE37534_UNTREATED_VS_ROSIGLITAZONE_TREATED_CD4_TCELL_PPARG1_AND_FOXP3_TRASDUCED_DN	MSigDB lists

-4.06	Ppargc1b (peroxisome proliferative activated receptor, gamma, coactivator 1 beta)	protein interactions
-4.03	GSE25123_WT_VS_PPARG_KO_MACROPHAGE_ROSIGLITAZONE_STIM_DN	MSigDB lists
-3.91	GSE37532_TREG_VS_TCONV_PPARG_KO_CD4_TCELL_FROM_VISCERAL_ADIPOSE_TISSUE_UP	MSigDB lists
-3.89	GSE25123_CTRL_VS_IL4_AND_ROSIGLITAZONE_STIM_PPARG_KO_MACROPHAGE_UP	MSigDB lists
-3.81	GSE37533_UNTREATED_VS_PIOGLIZATONE_TREATED_CD4_TCELL_PPARG2_AND_FOXP3_TRASDUCED_UP	MSigDB lists
-3.77	GSE5679_PPARG_LIGAND_ROSIGLITAZONE_VS_RARA_AGONIST_AM580_TREATED_DC_UP	MSigDB lists
-3.53	GSE25123_CTRL_VS_IL4_STIM_PPARG_KO_MACROPHAGE_UP	MSigDB lists
-3.45	GSE25123_CTRL_VS_IL4_AND_ROSIGLITAZONE_STIM_PPARG_KO_MACROPHAGE_DN	MSigDB lists
-3.36	GSE5679_CTRL_VS_PPARG_LIGAND_ROSIGLITAZONE_TREATED_DC_UP	MSigDB lists
-3.26	Ppargc1a (peroxisome proliferative activated receptor, gamma, coactivator 1 alpha)	protein interactions
-3.00	GSE24292_WT_VS_PPARG_KO_MACROPHAGE_UP	MSigDB lists

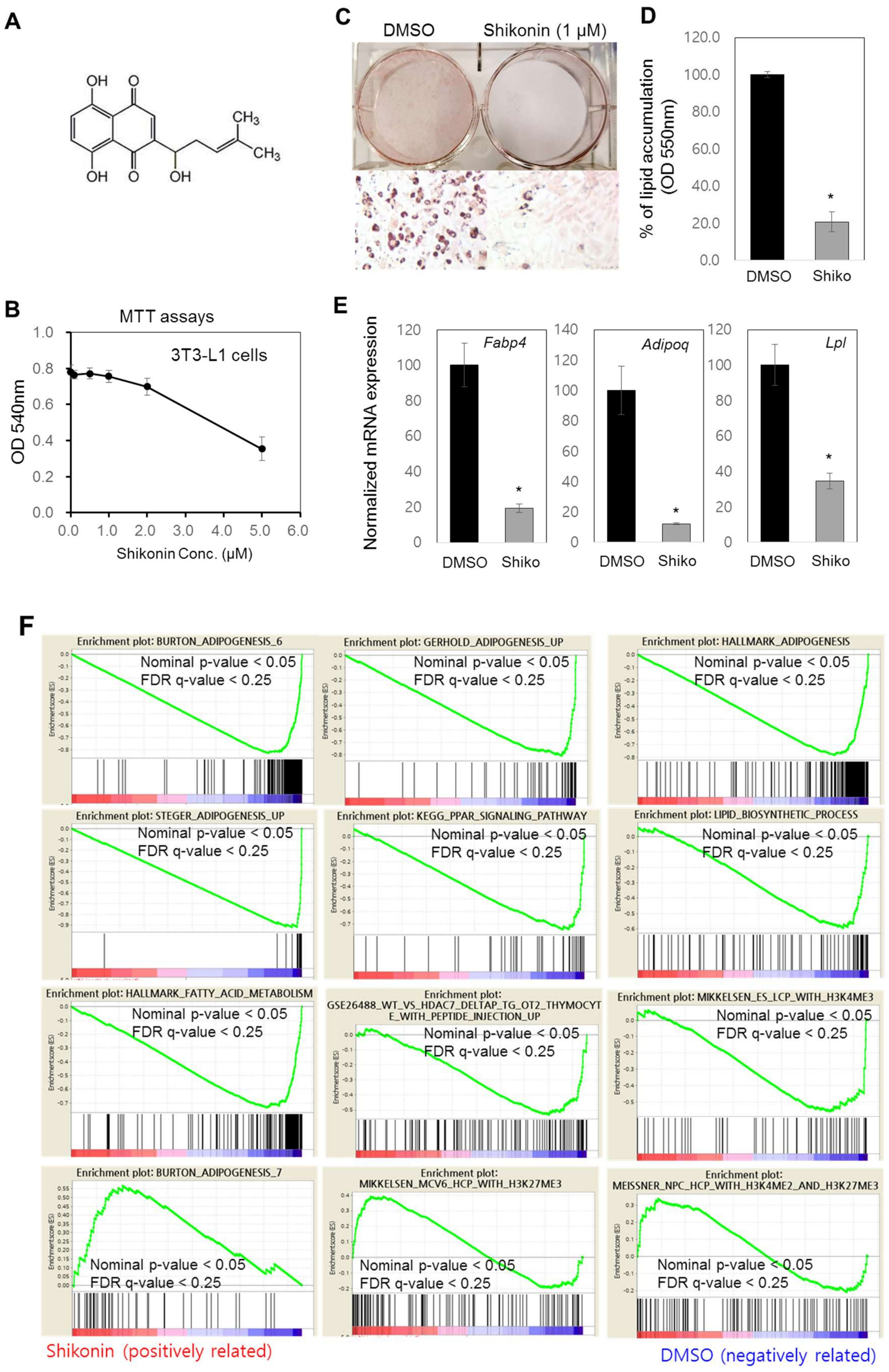
A
OH
O
CH3
CH3
OH
O
OH
B
MTT assays
3T3-L1 cells
OD 540nm
Shikonin Conc. (μM)
C
DMSO
Shikonin (1 μM)
D
% of lipid accumulation (OD 550nm)
DMSO
Shiko
E
Normalized mRNA expression
Fabp4
Adipoq
Lpl
DMSO
Shiko
F
Enrichment plot: BURTON_ADIPOGENESIS_6
Enrichment plot: GERHOLD_ADIPOGENESIS_UP
Enrichment plot: HALLMARK_ADIPOGENESIS
Enrichment plot: STEGER_ADIPOGENESIS_UP
Enrichment plot: KEGG_PPAR_SIGNALING_PATHWAY
Enrichment plot: LIPID_BIOSYNTHETIC_PROCESS
Enrichment plot: HALLMARK_FATTY_ACID_METABOLISM
Enrichment plot: GSE26488_WT_VS_HDAC7_DELTAP_TG_OT2_THYMOCYTE_WITH_PEPTIDE_INJECTION_UP
Enrichment plot: MIKKELSEN_ES_LCP_WITH_H3K4ME3
Enrichment plot: BURTON_ADIPOGENESIS_7
Enrichment plot: MIKKELSEN_MCV6_HCP_WITH_H3K27ME3
Enrichment plot: MEISSNER_NPC_HCP_WITH_H3K4ME2_AND_H3K27ME3
Nominal p-value < 0.05
FDR q-value < 0.25
Shikonin (positively related)
DMSO (negatively related)

Supplementary Figure S1. Anti-adipogenic effects of shikonin in 3T3-L1 cells. (**A**) Structure of shikonin. (**B**) Cytotoxicity of shikonin, measured by MTT assays. 3T3-L1 cells were grown in growth media as described in materials and method and plated at 500 cells/well in a 96-well tissue culture plate. Indicated concentrations of shikonin were treated and incubated for 72 h. 50 μL of MTT reagent (2 mg/mL) was added and incubated for 4 h. The MTT was dissolved with 0.1 mL of DMSO, and absorbance was read at 540 nm. (**C**, **D**) 3T3-L1 cells were differentiated and treated with DMSO or shikonin (1 μM). After differentiation for 8 days, lipid droplets in cells were fixed and stained with Oil Red O (**C**) and quantified at an OD of 500 nm (**D**). (**E**) Effects of shikonin on the mRNA expression of adipogenic genes (*Fabp4*, *Adipoq*, and *Lpl*). mRNA ex-pression, measured by RT-qPCR, was normalized to GAPDH expression and presented as a relative value to the DMSO control. Data are shown as means ± SDs for three independent experi-ments (*$p < 0.05$). (**F**) Gene set enrichment analysis (GSEA) of genes with altered expression after shikonin treatment. A nominal p-value < 0.05 and false discovery rate (FDR) q-value < 0.25 were considered to indicate statistical significance.

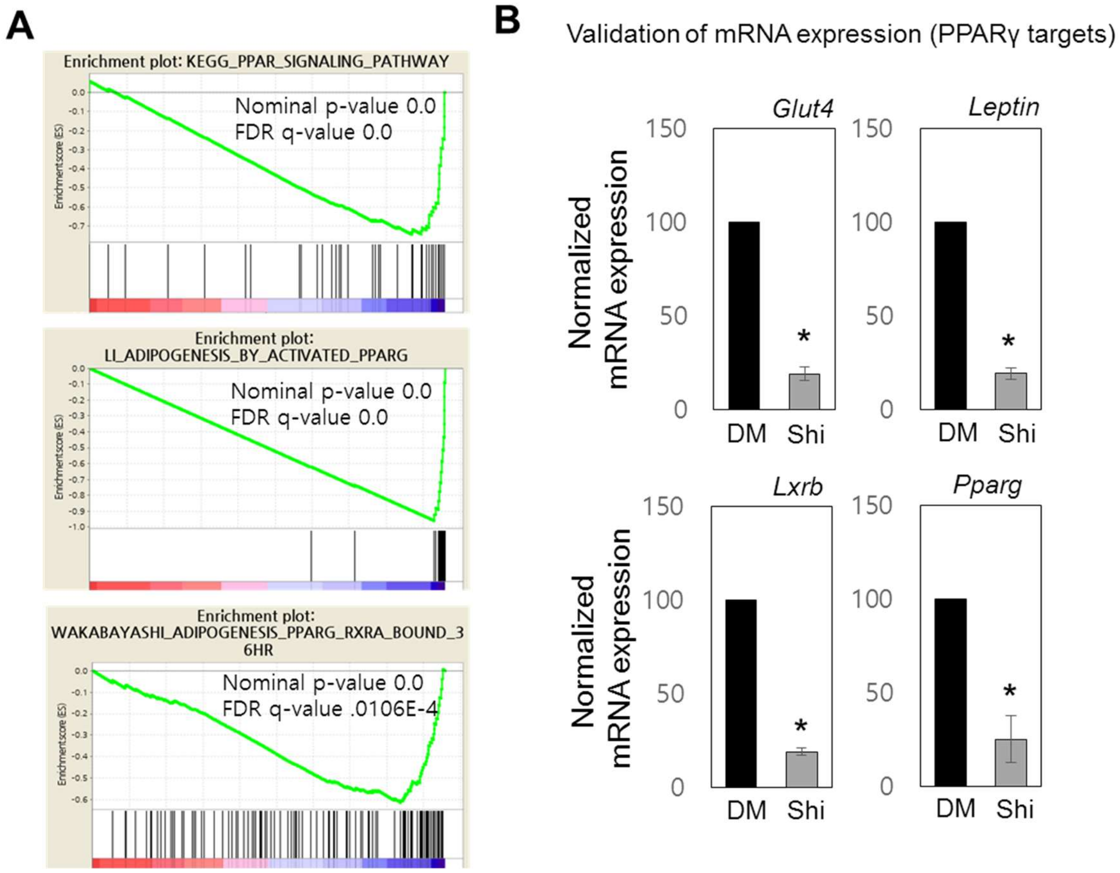

Supplementary Figure S2. Validation of RNA-seq data. (**A**) GSEA of KEGG_PPAR_SIGNALING_PATHWAY, LI_ADIPOGENESIS_BY_ACTIVATED_PPARG, and WAKABAYASHI_ADIPOGENESIS_PPARG_RXRA_BOUND_36HR. (**B**) Effects of shikonin on the mRNA expression of the PPARγ target genes *Glut4, Leptin, Lxrb, and Pparg*. 3T3-L1 cells were differentiated and treated with DMSO or shikonin. mRNA expression was measured by RT-qPCR and normalized to GAPDH expression. The mRNA expression level is presented relative to that of the DMSO control. Data are shown as means ± SDs for three independent experiments (*$p < 0.05$).

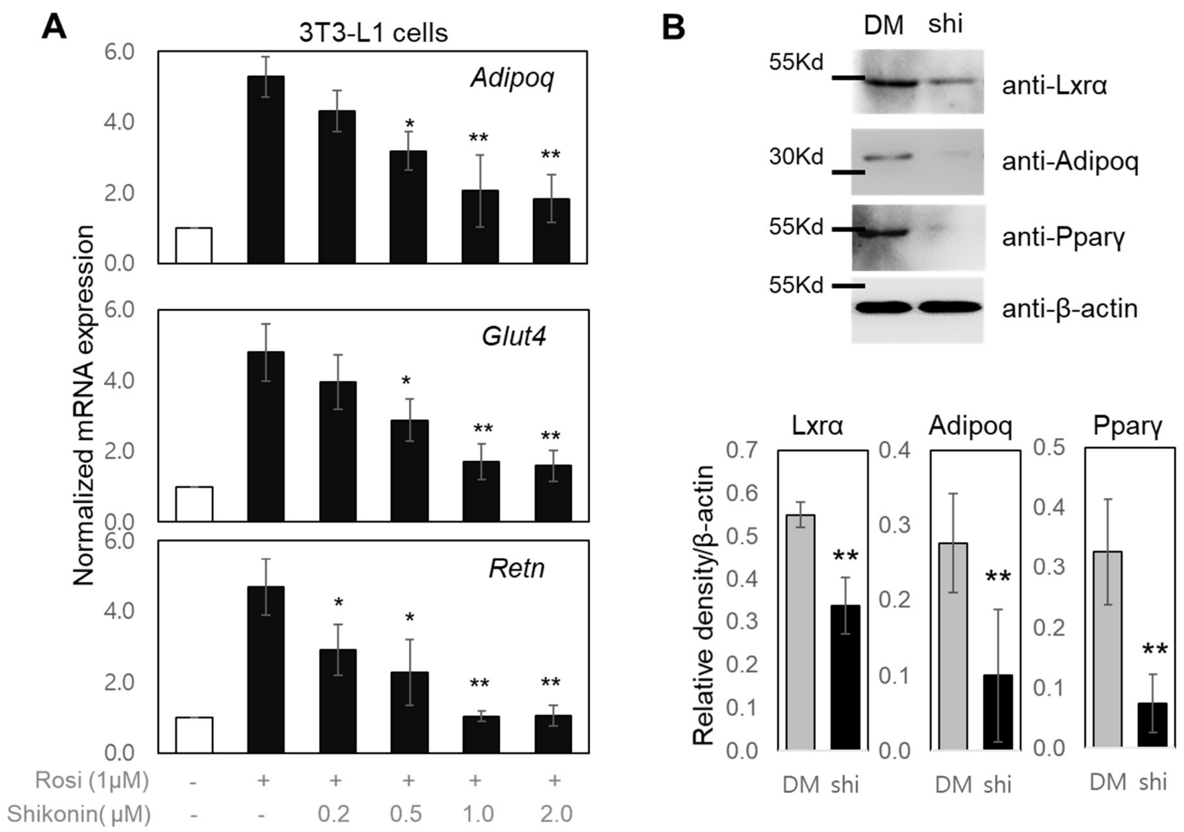

Supplementary Figure S3. Effect of shikonin on rosiglitazone-induced activation of the PPARγ target genes. (**A**) The mRNA expression of PPARγ target genes *Adipoq*, *Glut4*, and *Retn* was determined as described in Figure 2C. (**B**) Effect of shikonin on the protein expression of Lxrα, Adipoq, and PPARγ in 3T3-L1 cells. 3T3-L1 cells were differentiated upon DMSO or shikonin for 6 days. Lysates were separated by SDS-PAGE on 10 % gels, transferred to PVDFs membrane, and blotted with antibodies using indicated antibodies. Density were measured by ImageJ and normalized with density of anti-β-actin. Bars represent means ± SDs (*$p < 0.05$ and **$p < 0.01$).

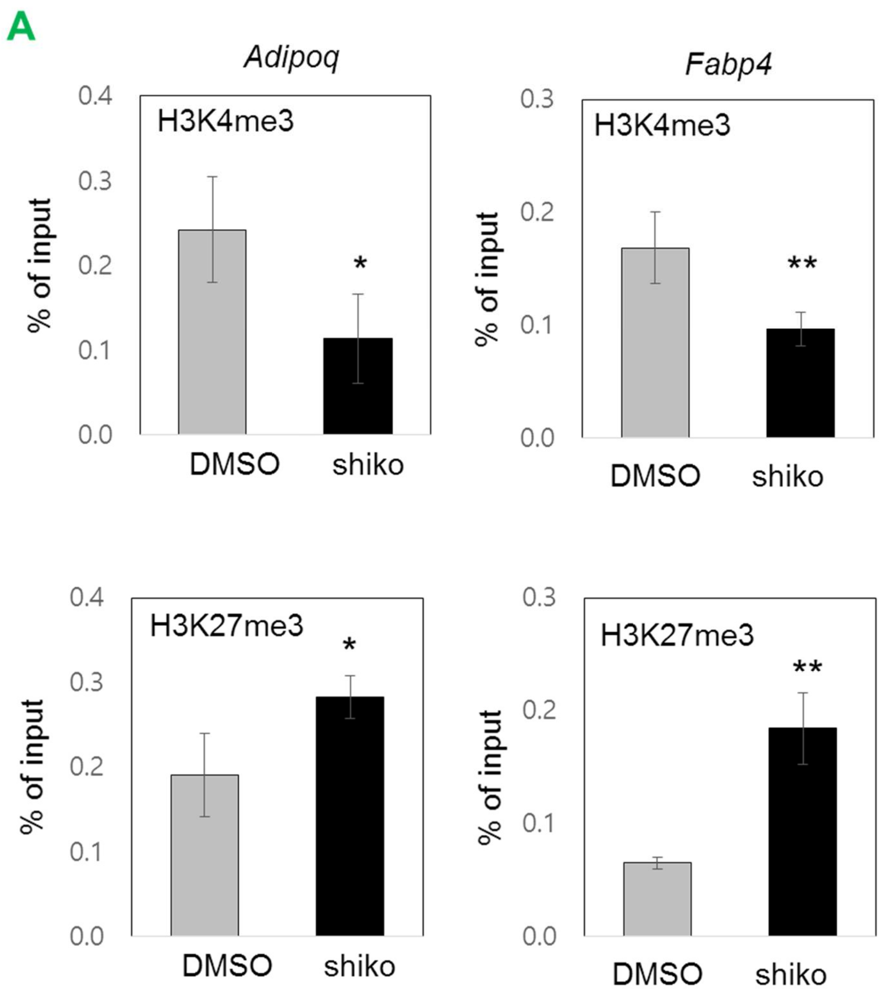

A
Adipoq
Fabp4
H3K4me3
H3K27me3
% of input
0.0
0.1
0.2
0.3
0.4
*
**
DMSO
shiko

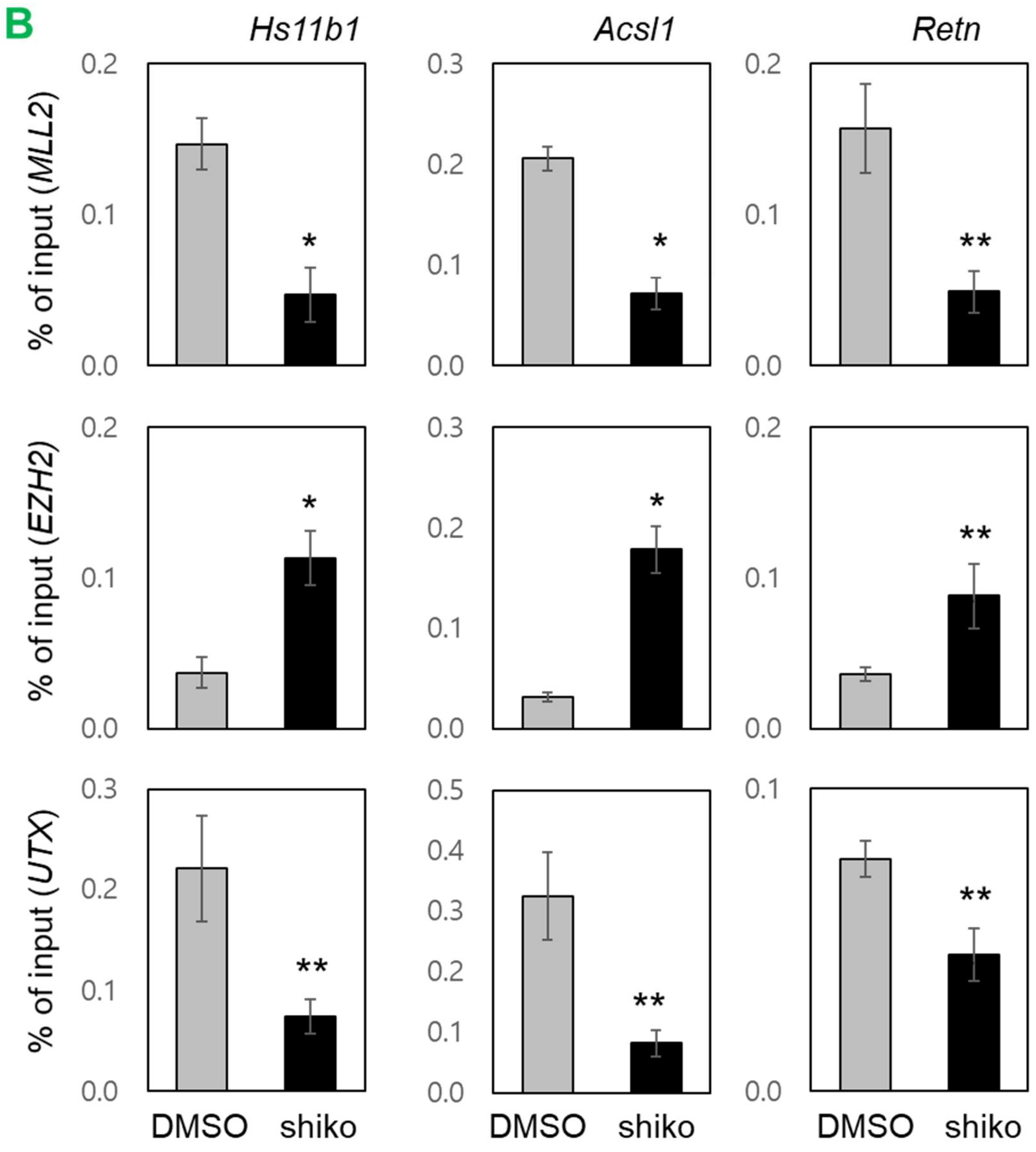

Supplementary Figure S4. Epigenetic regulation of PPARγ target genes by shikonin. ChIP assays were performed as described in Figure 4. (**A**) Two other PPARγ target genes, *Adipoq* and *Fabp4*, were used for assays. (**B**) ChIP assays using the indicated antibodies against MLL2, EZH2, and UTX. Binding occupancy was determined using qPCR and primer sets for the targeted promoters. Data are shown as means ± SDs for three independent experiments (*p < 0.05 and **p < 0.01).
